# Evolution Mechanism of Filtration Characteristics of Cement Grouting Materials in Sandy Medium

**DOI:** 10.3390/ma18102385

**Published:** 2025-05-20

**Authors:** Xiao Feng, Shilei Zhang, Zhenzhong Shi, Qingsong Zhang, Meiling Li, Wenda Yang, Wen Sun, Benao Hou

**Affiliations:** 1 School of Transportation Engineering, Shandong Jianzhu University, Jinan 250101, China; 2024155112@stu.sdjzu.edu.cn (Z.S.); 2024155129@stu.sdjzu.edu.cn (W.Y.); 2023155138@stu.sdjzu.edu.cn (W.S.); 2023155140@stu.sdjzu.edu.cn (B.H.); 2 Inner Mongolia Yin Chao Ji Liao Water Supply Co., Ltd., Xing’an League 137400, China; 13783654050@163.com (S.Z.); meiling250629@163.com (M.L.); 3 Institute of Geotechnical and Underground Engineering, Shandong University, Jinan 250061, China; zhangqingsong@sdu.edu.cn

**Keywords:** cement materials, sandy medium, grouting, darcy percolation, filtration effect, slurry diffusion

## Abstract

The seepage diffusion of cement grouting materials into a sandy medium is influenced by the skeleton’s adsorption and the pore channels’ tortuosity, resulting in heterogeneous retention of cement particles during migration. This study established a theoretical model for the filtration coefficient based on the mass balance equation and linear filtration law. Grouting tests were conducted to determine the density of the cement slurry at various diffusion positions, and the filtration coefficient was calculated using the theoretical model. Results indicate that the filtration coefficient varies dynamically along the diffusion distance rather than remaining constant. The surface filtration range of Grade 42.5 Portland Cement slurry in sample S1 is approximately 30 cm, with a final diffusion distance of 190 cm. In contrast, the surface filtration ranges for the 800 mesh superfine cement in S2 and the 1250 mesh superfine cement in S3 are less than 10 cm, resulting in final diffusion distances of 69 cm and 87 cm, respectively. This demonstrates that a longer surface filtration range in the sand sample corresponds to a farther final diffusion distance of the slurry. Additionally, a larger ratio of sand pore diameter to cement particle size results in a smaller filtration coefficient and a greater slurry diffusion distance. Under a constant water–cement ratio, smaller cement particle sizes are associated with decreased slurry fluidity, which reduces the diffusion of cement slurry within the sandy medium. The research findings provide valuable insights for designing borehole spacing in grouting treatment for sandy media.

## 1. Introduction

The design depth of urban rail transit lines is predominantly situated within shallow or sub-shallow layers, with subway tunnels frequently intersecting sandy strata, particularly in riverbanks and coastal cities [1,2,3]. The fragmented and water-saturated characteristics of the Quaternary sandy layer, combined with deteriorating groundwater seepage during construction, can lead to disasters, such as surface collapse and water–sand inrushes [4,5].

In the study of grouting within porous media, such as soft sandy layers, it is often assumed that the slurry disperses uniformly throughout the injected medium. When cement slurry (a suspended slurry) is employed for penetration grouting in porous media, the medium’s skeleton will intercept and adsorb cement particles. Larger particles encounter greater difficulty penetrating the pores and tend to accumulate in the surface layer, forming a “filter cake”, a phenomenon known as surface filtration. Smaller particles can penetrate the pores; however, due to skeleton adsorption and pore tortuosity, these particles experience non-uniform filtration retention during migration, a process called deep filtration.

Y.S. Yu et al. [6] examined the effects of filter layer height and particle size on filtration efficiency and fluid properties, concluding that an increase in filter layer height and a decrease in particle size enhance filtration efficiency but adversely affect pressure gradient reduction. Menghuo Chen et al. [7] investigated the angular variation of the seepage field in anisotropic porous media and analyzed the influence of anisotropy on the migration and deposition of nanoparticles. Zilong Zhou et al. [8] assert that when the mass fractal dimension of the porous medium is high, the deposition rate of cement particles decreases significantly with increasing diffusion distance. Binbin Ding et al. [9] proposed a strain-based deep filtration model and investigated the lattice type, lattice ratio, and particle trapping mechanisms associated with deep filtration. Y. Thomas Carraro et al. [10], Juncheng Qiao et al. [11], and Fei Ye et al. [12] investigated the external boundary effective pressure and the permeability empirical equation of porous media under filtration effects, yielding substantial experimental results. Jenchen Fan et al. [13] analyzed the results of indoor sand column tests using the rank correlation coefficient method and stepwise regression analysis, elucidating the effects of sand particle effective diameter and slurry viscosity on grouting time, grouting rate, and water output rate. H. Bayesteh et al. [14] conducted prototype tests on low-water-content clay, thoroughly discussing the influence of grouting technical parameters on slurry diffusion distance and revising the existing estimation formula for slurry diffusion distance. Xiaoli Liu et al. [15] simulated the diffusion process of Bingham fluid in fine sand layers under varying grouting pressure conditions using the discrete element method, establishing the relationship among grouting pressure, diffusion radius, and initial breakdown pressure. Almir Draganovic et al. [16] investigated the injectivity of three cement-based grouting materials, concluding that the maximum allowable opening for fracture injection is 75 μm. Fanlu Min et al. [17] employed a self-developed penetration device to conduct grouting tests and established a theoretical model for sealing the voids of the injected medium. Mohamed Shousha et al. [18] investigated the effects of grouting curtains on the seepage characteristics of small hydraulic structures, establishing a seepage equation influenced by these curtains through regression analysis, and found that the filtration reduction effect of the grouting curtain is 20% more effective than that of the upstream covering layer. Fang Hongyuan et al. [19] took sand layer particle size, dynamic water pressure, grouting pressure, and clay content as factors to study the dominant controlling conditions for the transportation and diffusion of polymer slurry in water-rich sand layers. Deng Fucheng et al. [20] revealed the permeability evolution laws of gravel-pack layers under sand intrusion and hydrate formation conditions based on the synergistic plugging mechanism of medium sand and hydrate within the gravel-pack layer. Ying Cui et al. [21] prepared a DS grouting material for water-rich, silty, fine sand strata using sodium silicate as the base material and diacid ester as the curing agent, featuring an adjustable setting time, high strength, durability, and exemplary performance in tunnel reinforcement. Sun Xu et al. [22] conducted filtration loss tests with different bentonite–ultrafine cement slurry mixtures and performed a microstructural analysis of the filtration cakes, concluding that the bentonite content and the distribution of its hydrated colloids determine the filtration performance. Tongqiang Xiao et al. [23] analyzed the effects of water-reducing agent content on ultrafine cement slurry’s viscosity and bleeding rate. Dongyue Zhang et al. [24] prepared grouted specimens with two water–cement ratios and four superfine cement contents using mercury intrusion tests, fragment fractal dimension analysis, and SEM, finding that increasing the content enhances strength and reduces porosity and fractal dimension. Yuya Sakai et al. [25] used mercury intrusion porosimetry to conduct a three-dimensional diffusion simulation of the pore structure in cementitious materials, and they evaluated the diffusion coefficient of cement paste within these materials. Huang Liwei et al. [26] conducted pressure filtration grouting tests, tested the strengthening strength of the consolidated body under different water–cement ratios and pressure filtration intensities, and established a strengthening strength model for ultrafine cement. Abou-Chakra et al. [27] proposed a multiscale model based on microstructural characteristics for cement-based materials with recycled rubber aggregates to predict diffusion properties considering the interface transition zone (ITZ), which was validated by experiments. Chuen-Ul Juang et al. [28] developed a green grouting material using a low water-to-binder ratio, 20% slag/fly ash, 2–3% water reducer, and 60–70% sand. The material exhibited excellent durability, and the regression analysis was reliable. Li Zhipeng et al. [29] addressed the issue of the lack of a theoretical basis for grouting design in sand layers, proposing a design method to quantitatively determine the grouting mode, the grouting range, and the reinforcement effect and establishing a theoretical model for fracture–compaction grouting. Feng Xiao et al. [30] prepared a novel slurry by adding expansion agents, water reducers, and accelerating agents. Tests showed that its fluidity, strength, and expansion rate improved, and field application significantly enhanced the stratum’s impermeability.

Based on the above research, existing studies have predominantly focused on the injectability assessment of cement slurry, often utilizing the ratio of cement particle size to that of the medium skeleton particles. However, the surface filtration effect and final diffusion distance of cement slurry in the sandy medium have been neglected. Additionally, in the experimental studies, particle filtration quality was determined solely by calculating the mass difference before and after grouting, concluding that the filtration coefficient is a constant value. However, the variation of the filtration coefficient along the diffusion distance was not investigated.

Therefore, this study establishes a time-dependent filtration–diffusion coupling model to quantitatively characterize grout performance degradation in sandy media. The model is grounded in the following assumptions: complete saturation of the sand sample, Newtonian fluid behavior of the cement slurry, and a planar laminar flow regime of the grout. The proposed framework dynamically links time-varying filtration parameters to grout diffusion behavior by combining the mass balance equation and the linear filtration law. Through grouting tests, the density values of the cement slurry at various diffusion distances were obtained, the filtration coefficient was calculated based on the coupling model, and the variation trend of the filtration coefficient was analyzed. The research results can be used to obtain the surface plugging time and the final diffusion distance of the slurry in the sandy medium, thereby guiding the design of the borehole spacing in the grouting treatment of sandy strata.

## 2. Establishment of the Mathematical Model

In this study, the sandy medium is assumed to be fully saturated, and the ordinary Portland Cement slurry, as a particle suspension, exhibits the characteristics of a Newtonian fluid. The essence of grouting pressure lies in its influence on the slurry flow rate (i.e., slurry velocity). Because grouting pump equipment typically uses the flow rate as a control parameter, this study selects slurry velocity as the control indicator. Furthermore, by setting the Reynolds number (which is closely related to slurry velocity), the slurry is ensured to exhibit plane parallel laminar flow in the sandy medium, thereby satisfying the Darcy flow condition (Re ≤ 1).

A particle (with mass but no size or shape) with mass *m*_i_ is selected for analysis in the Cartesian coordinate system. At time *t*_1_, the particle is at point *x*_1_ and has a mass *m*_i_ (*x*_1_, *t*_1_). After a time interval of Δ*t*, the particle moves to point *x*_2_ = *x*_1_ + *v*Δ*t* with a velocity of *v*, and its mass is expressed as *m*_i_ (*x*_2_, *t*_2_) = *m*_i_ (*x*_1_ + *v*Δ*t*, *t*_1_ + Δ*t*).

Thus, the material derivative of mass *m*_i_ is given by

(1) $$\frac{dm_{i}}{dt}=\underset{\Delta t\to 0}{\lim}\frac{m_{i}(x_{2},t_{2})-m_{i}(x_{1},t_{1})}{\Delta t}=\underset{\Delta t\to 0}{\lim}\frac{m_{i}(x_{1}+v\Delta t,t_{1}+\Delta t)-m_{i}(x_{1},t_{1})}{\Delta t}$$

Expanding using the Taylor series yields

(2) $$m_{i}(x_{1}+v\Delta t,t_{1}+\Delta t)=m_{i}(x_{1},t_{1})+\frac{\partial m_{i}}{\partial t}\cdot \Delta t+\left(v\cdot \nabla \right)m_{i}\cdot \Delta t+\frac{1}{2}\left[\frac{\partial^{2}m_{i}}{\partial t^{2}}+2v\cdot \nabla \left(\frac{\partial m_{i}}{\partial t}\right)+{\left(v\cdot \nabla \right)}^{2}m_{i}\right]\cdot \Delta t^{2}+O\left(\Delta t^{3}\right)$$

(3) $$\frac{dm_{i}}{dt}=\frac{\partial m_{i}}{\partial t}+\left(v\cdot \nabla \right)m_{i}$$

To investigate the influence of the filtration effect on the movement of cement particles in the sandy medium, we assume that the retained cement particles become part of the medium skeleton, which means that the filtered cement particles participate in its formation. Consequently, filtration can be regarded as a process in which the cement slurry loses the cement particles, and the medium skeleton obtains them.

Based on the mass balance Equation (3), the mass balance expression for cement particles is as follows

(4) $$-\mu =\frac{\partial (\rho_{c}n_{c})}{\partial t}+(v_{c}\cdot \nabla )(\rho_{c}n_{c})$$

The mass balance Equation for water can be expressed as

(5) $$0=\frac{\partial (\rho_{w}n_{w})}{\partial t}+(v_{w}\cdot \nabla )(\rho_{w}n_{w})$$

The mass balance Equation for the sandy medium skeleton is expressed as

(6) $$\mu =\frac{\partial (\rho_{s}n_{s})}{\partial t}+(v_{s}\cdot \nabla )(\rho_{s}n_{s})$$

In the Equations, *ρ*_c_ represents the density of cement particles (2.80–3.15 g/cm^3^); *ρ*_w_ denotes the density of water (g/cm^3^); ρ_s_ represents the density of the sandy medium skeleton (2.65–2.76 g/cm^3^); *n*_c_ indicates the volume percentage content of cement particles per unit volume (dimensionless); *n*_w_ denotes the volume percentage content of water per unit volume (dimensionless); *n*_s_ is the volume percentage content of the sandy medium skeleton per unit volume (dimensionless); *t* represents time (s); *v*_c_ denotes the velocity of cement particles (cm/s); *v*_w_ denotes the velocity of water (cm/s), where *v*_w_ = *v*_c_; *v*_s_ refers to the velocity of the medium skeleton (cm/s), where *v*_s_ = 0; and *μ* denotes the mass exchange coefficient, representing the mass of cement particles filtered out per unit time and unit volume (g·cm^−3^·s^−1^).

Assuming that the pores of the sandy medium are saturated with cement slurry and that the skeleton density of the medium is approximately equal to the density of the cement particles (*ρ*_s_ = *ρ*_c_), we can express the medium porosity as

(7) $$n=n_{w}+n_{c}=1-n_{s}$$

In the Equation, n represents the porosity of the sandy medium (dimensionless).

Because *v*_s_ = 0 cm/s, this can be derived by integrating Equation (7) with Equation (6).

(8) $$\frac{\partial \left[\rho_{s}n_{s}\right]}{\partial t}=\mu$$

(9) $$\frac{\partial \left(1-n\right)}{\partial t}=\frac{\mu }{\rho_{s}}$$

(10) $$\frac{\partial n}{\partial t}=-\frac{\mu }{\rho_{c}}$$

By the linear filtration law [31,32],

(11) $$\mu =\rho_{c}n_{0}\lambda f\left(\delta \right)$$

In the Equation, the scalar μ denotes the mass exchange coefficient, representing the mass of cement particles filtered out per unit time and unit volume, and *δ* indicates the volume fraction of cement particles within the pores, $\delta =\frac{n_{c}}{n_{0}}$.

Function f should comply with the physical condition *f* (0) = 0. Observing that, generally, *δ* << 1, Equation (11) may be simplified by adopting the first order of the Taylor expansion of f concerning *δ* [31,32]

(12) $$f\left(\delta \right)\approx \delta ,\mu =\rho_{c}n_{0}\lambda \delta$$

(13) $$\delta \left(x,t\right)=\frac{\rho_{g}-\rho_{w}}{\rho_{c}-\rho_{w}}=\left\{\begin{array}{l} \frac{1}{1+\left(\frac{1}{\varphi }-1\right)\exp\left(\frac{\lambda x}{n_{0}v_{0}}\right)}\;\;\left(0\leq x\leq v_{0}t\right)\\ 0\;\;\;\;\;\;\;\;\;\;\;\;\;\;\;\;\;\;\;\;\;\;\;\;\;\;\;\;\;\;\;\;\;\;\;\;\;\;\;\;\;\;\;\;\;\left(x>v_{0}t\right) \end{array}\right.\;$$

In the Equation, *λ* denotes the filtration coefficient (s^−1^); *n*_0_ represents the initial porosity of the sandy medium (dimensionless); *δ* indicates the volume fraction of cement particles within the pores (dimensionless); *ρ*_g_ represents the density of the cement slurry (g/cm^3^); *φ* denotes the volume fraction of cement particles within the cement slurry (dimensionless); *x* indicates the slurry diffusion distance (cm); and *v*_0_ refers to the velocity of the cement slurry (cm/s).

From Equations (12) and (13), it can be inferred that

(14) $$\lambda =\frac{\mu }{\rho_{c}n_{0}\delta }=\ln\left[\left(\frac{\rho_{c}-\rho_{w}}{\rho_{g}-\rho_{w}}-1\right)\times \frac{\varphi }{1-\varphi }\right]\times \frac{n_{0}v_{0}}{x}\;\;\;\;\;\;\;\;\;\;\;\;\;\;\;\;\;\left(0\leq x\leq v_{0}t\right)$$

Consequently, using Equation (14), one can calculate the filtration coefficient at various diffusion distances by utilizing the density of the cement slurry, the volume fraction of cement particles within the slurry, the initial porosity of the sandy medium, and the flow velocity of the cement slurry.

The boundary condition for the porosity of the sandy medium is as follows

(15) $$n\left(x,t\right)=n_{0}\;\;\mathrm{at}\;\;t=0$$

(16) $$n\left(x,t\right)=n_{0}\;\;\mathrm{at}\;\;x>v_{0}t$$

Substituting Equations (12) and (13) into Equation (10) and combining with Equations (15) and (16), it can be derived that

(17) $$n=\left\{\begin{array}{l} n_{0}-\lambda \left(t-\frac{x}{v_{0}}\right)\left[\frac{1}{1+\left(\frac{1}{\varphi }-1\right)\exp\left(\frac{\lambda x}{n_{0}v_{0}}\right)}\right]\;\left(0\leq x\leq v_{0}t=L\right)\\ n_{0}\;\;\;\;\;\;\;\;\;\;\;\;\;\;\;\;\;\;\;\;\;\;\;\;\;\;\;\;\;\;\;\;\;\;\;\;\;\;\;\;\;\;\;\;\;\;\;\;\;\left(x>v_{0}t\right) \end{array}\right.\;$$

At *x* = 0 cm (the initial interface of the slurry entering the sandy medium), when *n* = 0, it indicates that the pore channels of the sandy medium are entirely obstructed, resulting in

(18) $$n=\;n_{0}-\lambda \left(t-\frac{x}{v_{0}}\right)\left[\frac{1}{1+\left(\frac{1}{\varphi }-1\right)\exp\left(\frac{\lambda x}{n_{0}v_{0}}\right)}\right]=n_{0}-\lambda t\varphi =0$$

Therefore, we arrive at

(19) $$\lambda =\frac{n_{0}}{t\varphi }$$

Consequently, Equation (19) can be used to derive the filtration coefficient of the sand medium’s initial interface by utilizing the sandy medium’s initial porosity, the volume fraction of cement particles within the cement slurry, and the time when the sand medium’s initial interface (*x* = 0 cm) is completely blocked.

## 3. Grouting Model Experiment

### 3.1. Purpose of the Experiment

The grouting test of plane parallel laminar flow is designed to study the change in slurry density along the diffusion distance when the cement slurry flows in parallel in different sand mediums. 

### 3.2. Test Materials

#### 3.2.1. Sandy Medium

According to Sand for Construction, natural sand is defined as rock particles that are naturally formed and artificially mined and screened with a particle size smaller than 4.75 mm. This includes river sand, lake sand, mountain sand, and desalted sea sand, excluding soft and weathered rock particles. The fineness modulus *M*_x_ can classify natural sand into three categories, coarse sand (*M*_x_ = 3.1–3.7), medium sand (*M*_x_ = 2.3–3.0), and fine sand (*M*_x_ = 1.6–2.2), calculated according to Equation (20) [33]

(20) $$M_{x}=\frac{(A_{2}+A_{3}+A_{4}+A_{5}+A_{6})-5A_{1}}{100-A_{1}}$$

Specifically, *A*_1_, *A*_2_, *A*_3_, *A*_4_, *A*_5_, and *A*_6_ denote the cumulative sieve residue percentages for 4.75 mm, 2.36 mm, 1.18 mm, 0.60 mm, 0.30 mm, and 0.15 mm sieves, respectively.

According to the Standard for Geotechnical Testing Method, three sand samples were selected for sieve analysis. The particle gradation of the sand samples is presented in Table 1, and the structural parameters of the sand samples are detailed in Table 2.

Note: (1) *D*_0_ represents the effective diameter of the particles, defined as the average value of all particle diameters [33]


(21) $$D_{0}=\sum_{i=1}^{n}\frac{D_{i}q_{i}}{100}$$

In the Equation, *D*_0_ denotes the effective diameter of the sandy medium (mm), *D*_i_ represents the screening diameter of the sandy medium (mm), and *q*_i_ signifies the percentage associated with the screening diameter (dimensionless).

(2) *d*_0_ is the pore diameter [33]:

For a homogenized spherical granular medium

(22) $$d_{01}=0.855D_{0}\frac{{n_{0}}^{0.725}}{{\left(1-n_{0}\right)}^{0.5}}$$

For a uniform sand medium

(23) $$d_{02}=1.25n_{0}D_{0}$$

For quartz sand with a particle size ranging from 1 mm to 3.15 mm

(24) $$d_{03}=0.84n_{0}D_{0}+0.13$$

#### 3.2.2. Cement Slurry

The grouting test used Grade 42.5 Portland Cement, 800 mesh superfine cement, and 1250 mesh superfine cement. The average particle size *d*_50_ of Grade 42.5 Portland Cement measured using the TZC-2 automatic particle size analyzer is 17 μm, while the 800 mesh superfine cement is 7 μm, and the 1250 mesh superfine cement is 4 μm. The performance parameters of each cement slurry are presented in Table 3.

### 3.3. Test Scheme

To investigate the variation in slurry density and diffusion distance of cement slurry within a sandy medium under filtration effects and elucidate the interaction mechanism between surface filtration and deep filtration, grouting tests utilizing different types of cement slurries were conducted on three distinct sand samples. The testing scheme is presented in Table 4.

### 3.4. Test Process

**Figure 1:**
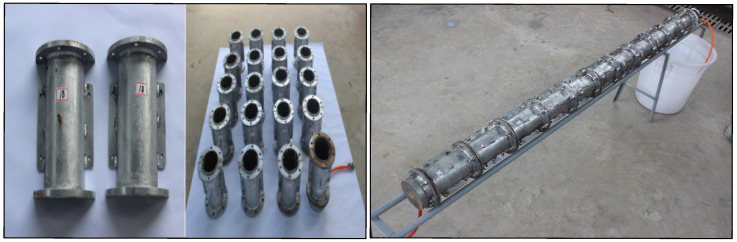
Cylindrical steel pipes.

**Figure 2:**
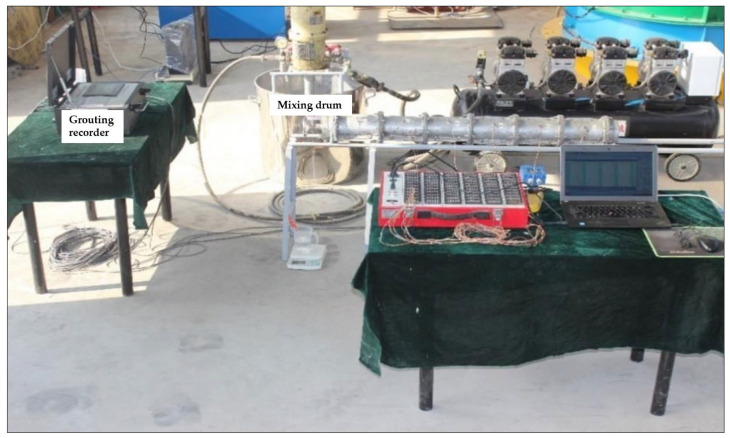
Grouting test system.

The grouting test system comprises a grout input system, a planar parallel flow device, and a monitoring system. The planar parallel flow device includes an inlet chamber, cylindrical steel pipes, and an outlet chamber. Each single cylindrical steel pipe has an inner diameter of 100 mm, a height of 200 mm, and a wall thickness of 5 mm, and 20 such pipes were designed. The cylindrical steel pipes are illustrated in Figure 1, while the overall grouting test system is depicted in Figure 2.

The single cylindrical steel pipe is divided into symmetrical sections to facilitate disassembly and coring. The inner wall of each steel pipe is abraded and uniformly coated with a lubricant to eliminate edge effects. A small hole is opened in the middle of the steel pipe, and a sealing joint and a one-way valve are installed to collect the slurry flowing out through the small hole. The single cylindrical steel pipes exhibit high strength and rigidity, preventing deformation.

In each group of grouting tests, the effluent slurry was collected at different positions of the parallel flow device. The volume and mass of the slurry were measured to determine its density. Then, the cement slurry with the same density was prepared in the laboratory, and the water–cement ratio of the effluent slurry could be obtained through the inverse method so that the mass fraction and volume fraction of cement particles in the slurry at different positions could be calculated.

### 3.5. Experimental Results

When the Reynolds number (Re) of the cement grout flowing in a sandy medium is Re ≤ 1, it indicates that the cement grout exhibits plane parallel flow [33], which satisfies the application premise of the established mathematical model.

(25) Re=100×ρgv0d10μ1

In the equation, *v*_0_ refers to the velocity of the cement slurry (cm/s); *d*_10_ is the effective particle size of solid particles in a sandy medium (cm); and *μ*_1_ is the dynamic viscosity of cement slurry (mPa·s).

**Figure 3:**
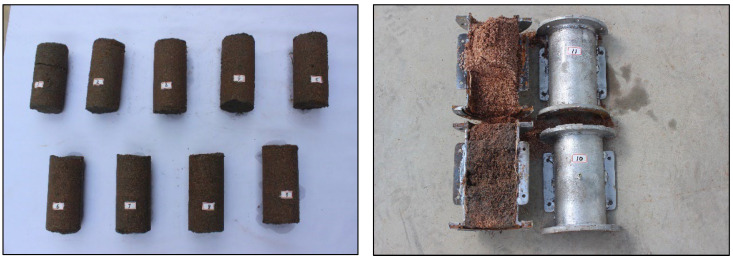
S1 sand sample—Grade 42.5 Portland Cement experimental results.

For the S1 sand sample, *n*_0_ = 0.388 and *d*_10_ = 0.037 cm; the density of Grade 42.5 Portland Cement with a water–cement ratio of 1:1 is *ρ*_g_ = 1.45 g/cm^3^, *φ* = 0.275, and *μ*_1_ = 4.53 mPa·s; and the initial velocity of the slurry is 0.85 cm/s, with a sampling duration of 20 s at each point. Through Equation (25), the Reynolds number is calculated to be 1.0, indicating that the Grade 42.5 Portland Cement slurry exhibits plane parallel flow in the S1 sand sample. In addition, the grouting test lasts about 224 s, and the slurry’s initial setting time is about 53,760 s (Table 3, 896 min), indicating that the slurry is a Newtonian fluid during the test. Detailed test results are presented in Table 5 and Figure 3.

**Figure 4:**
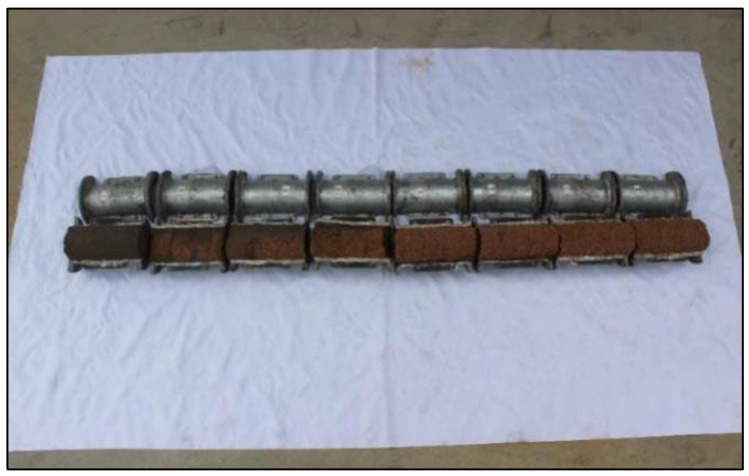
S2 sand sample—800 mesh superfine cement experimental results.

For the S2 sand sample, *n*_0_ = 0.338 and *d*_10_ = 0.037 cm; the density of 800 mesh superfine cement with a water–cement ratio of 1:1 is *ρ*_g_ = 1.46 g/cm^3^, *φ* = 0.27, and *μ*_1_ = 5.87 mPa·s; and the initial velocity of the slurry is 0.85 cm/s. The sampling time of each point is 20 s, but the sampling time at 50 cm is 10 s. Through Equation (25), the Reynolds number is calculated to be 0.79, indicating that the 800 mesh superfine cement slurry exhibits plane parallel flow in the S2 sand sample. In addition, the grouting test lasts about 82 s, and the slurry’s initial setting time is about 45,780 s (Table 3, 763 min), indicating that the slurry is a Newtonian fluid during the test. Detailed experimental results can be found in Table 6 and Figure 4.

**Figure 5:**
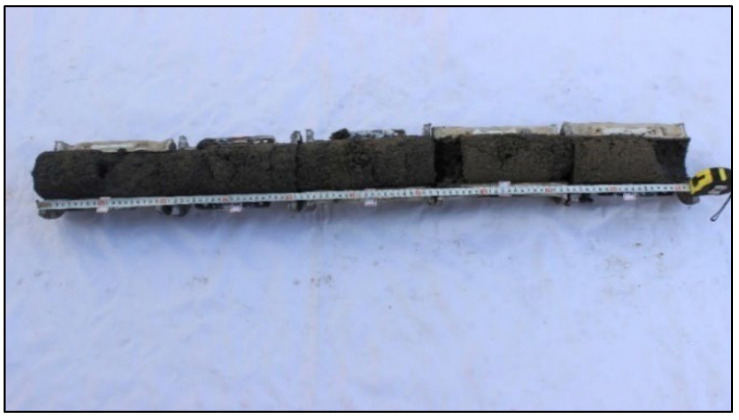
S3 sand sample—1250 mesh superfine cement experimental results.

For the S3 sand sample, *n*_0_ = 0.329 and *d*_10_ = 0.034 cm; the density of 1250 mesh superfine cement with a water–cement ratio of 1:1 is *ρ*_g_ = 1.48 g/cm^3^, *φ* = 0.26, and *μ*_1_ = 6.98 mPa·s; and the initial velocity of the slurry is 0.85 cm/s, with a sampling duration of 20 s at each point. Through Equation (25), the Reynolds number is calculated to be 0.61, indicating that the 1250 mesh superfine cement slurry exhibits plane parallel flow in the S3 sand sample. In addition, the grouting test lasts about 103 s, and the slurry’s initial setting time is about 37,800 s (Table 3, 630 min), indicating that the slurry is a Newtonian fluid during the test. Detailed experimental results can be found in Table 7 and Figure 5.

Comprehensive analysis of Table 5, Table 6 and Table 7 summarizes the filtration coefficients (*λ*) for S1, S2, and S3 sand samples, measured as *λ* = 1.24–18.17 × 10⁻^3^ s⁻^1^, 3.29–8.86 × 10⁻^3^ s⁻^1^, and 1.86–7.15 × 10⁻^3^ s⁻^1^, respectively. These ranges reflect the dynamic evolution of filtration characteristics under varying particle gradation and cement slurry penetration depths. Specifically, S1 exhibits the widest *λ* range (1.24–18.17 × 10⁻^3^ s⁻^1^), attributed to its heterogeneous particle size distribution, which amplifies localized clogging effects during grout diffusion. S2 and S3 show narrower *λ* ranges due to their more uniform gradation, aligning with the model’s prediction of reduced filtration variability in homogeneous media.

## 4. Test Data Analysis

### 4.1. Variation of Filtration Coefficient Along Slurry Diffusion Distance

Analysis of Table 5, Table 6 and Table 7 indicates that the density of the cement slurry decreases with increasing diffusion distance within the sandy medium, suggesting that the filtration coefficient diminishes from a higher to a lower value along this distance. However, previous studies have concluded that the filtration coefficient remains constant, as detailed in Table 8.

Based on the data presented in Table 8, the following can be concluded.

(1) Most previous studies have used artificial suspended particles and porous media with uniform particle sizes and smooth surfaces. In contrast, this study employs natural sand samples, Portland Cement, and superfine cement, and there is an essential difference between this study and previous studies regarding the experiment’s subjects.

(2) Previous studies determined the corresponding filtration coefficient *λ* by measuring the mass difference of the porous media before and after the experiment. This study monitored parameters like slurry density and flow rate at various diffusion distances of sand samples, with Equation (14) employed to calculate the theoretical value of *λ*.

(3) The average filtration coefficient for each sand sample is adopted as the evaluation standard; specifically, the average filtration coefficient of the S1 sand sample is *λ* = 4.46 × 10^−3^s^−1^, the S2 sand sample is *λ* = 4.96 × 10^−3^s^−1^, and the S3 sand sample is *λ* = 3.59 × 10^−3^s^−1^. Although the experimental materials in this study differ from those used in previous research, both sets of test results fall within the same order of magnitude and exhibit a high degree of similarity. This suggests that it is reasonable to quantitatively calculate the filtration coefficient based on the model of the filtration coefficient under the condition of plane parallel laminar flow.

(4) According to the grouting test, the filtration coefficient of the S1 sand sample is *λ* = 1.24–18.17 × 10^−3^s^−1^, the S2 sand sample is *λ* = 3.29–8.86 × 10^−3^s^−1^, and the S3 sand sample is *λ* = 1.86–7.15 × 10^−3^s^−1^. The overall filtration coefficient range in this study spans 1.24–18.17 × 10^−3^s^−1^. Existing studies report values like *λ* = 0.99 × 10^−3^s^−1^ [35], *λ* = 35.0–47.88 × 10^−3^s^−1^ [36], *λ* = 0.52 × 10^−3^s^−1^ [37], and *λ* = 0.84 × 10^−3^s^−1^ [38], which differ from our findings. However, most existing data fall within the range identified in this study. Notably, previous experiments employed porous media and suspensions containing plastic balls [35], glass microspheres [36], colibacillus [37], and iron hydroxide [38]. This contrast highlights the critical role of particle morphology (roughness, angularity) in filtration evolution, as idealized media lack the inherent mechanisms of filtration, adsorption, and deposition in natural sand–cement systems.

### 4.2. Variation Law of Surface Filtration

Based on the sandy medium’s original porosity *n*_0_, the volume fraction *φ* of cement particles in the slurry, and the grouting time t, the filtration coefficient *λ* when all of the pore channels on the surface of the sand medium are fully obstructed can be calculated through Equation (19). For the S1 sand sample, the total grouting duration of 224 s results in a filtration coefficient of 6.3 × 10^−3^s^−1^, indicating that at this value, all pores at the surface of the sand medium are obstructed. Similarly, for the S2 sand sample with a total grouting time of 82 s, the filtration coefficient upon complete blockage is 15.27 × 10^−3^s^−1^. Additionally, for the S3 sand sample with a total grouting duration of 103 s, its corresponding filtration coefficient when entirely blocked is 12.29 × 10^−3^s^−1^.

**Figure 6:**
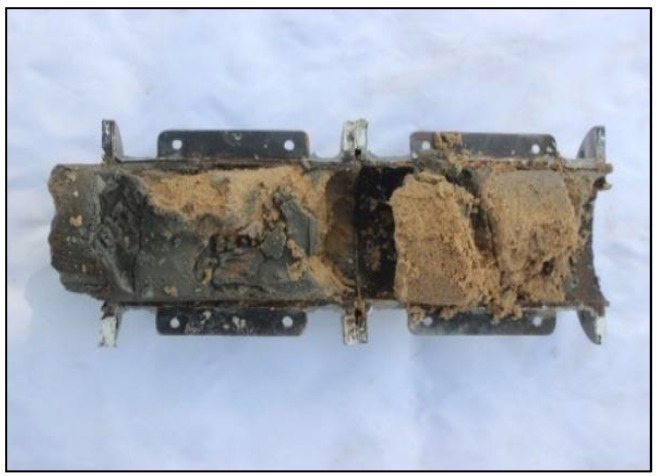
Schematic diagram of surface filtration.

Surface filtration occurs when the pore channel of the sandy medium is fully obstructed at a specific position. The cement slurry continues to enter the sand medium under grouting pressure and squeezes the medium skeleton, resulting in excessive siltation of cement particles. This phenomenon is illustrated in Figure 6. For instance, the filtration coefficient of the S1 sand sample at which all pore channels are fully obstructed is 6.3 × 10^−3^s^−1^, corresponding to a diffusion depth of approximately 30 cm; consequently, the surface filtration range extends from 0 to 30 cm. In contrast, for the S2 sand sample, when pore channels are entirely blocked, the filtration coefficient is 15.27 × 10^−3^s^−1^, with a surface filtration range extending from 0 to 10 cm. Similarly, for the S3 sand sample, its filtration coefficient upon complete blockage of pore channels is 12.29 × 10^−3^s^−1^, and it also exhibits a surface filtration range from 0 to 10 cm.

Furthermore, the range of surface filtration for the S1 sand sample extends from 0 to 30 cm, with a slurry diffusion distance of 190 cm. In contrast, the ranges of surface filtration for the S2 and S3 sand samples are from 0 to 10 cm, corresponding to slurry diffusion distances of 69 cm and 87 cm, respectively. It is concluded that the longer the distance of surface filtration in the sand sample, the farther the final diffusion distance of the slurry.

### 4.3. The Influence of the Ratio of Pore Diameter to Cement Particle Size on Filtration Performance

The average particle size *d*_50_ of Grade 42.5 Portland Cement is 17 μm, while the average particle sizes *d*_50_ for 800 mesh superfine cement and 1250 mesh superfine cement are 7 μm and 4 μm, respectively. By referencing Table 2, the ratio of the pore diameter of the sand medium to the cement particle size can be obtained. Additionally, values for the filtration coefficient at various locations are presented in Table 9 for comprehensive analysis.

Analysis of Table 9 indicates that at specific diffusion locations, the filtration coefficient of the S1 sand sample is the highest, followed by the S2 sand sample. In contrast, the S3 sand sample exhibits the lowest value. For instance, at a distance of 10 cm, the *λ* for the S1 sand sample is 18.17 × 10^−3^s^−1^, for the S2 sand sample, it is 8.86 × 10^−3^s^−1^, and for the S3 sand sample, it is 7.15 × 10^−3^s^−1^.

Analysis indicates that the S1 sand sample’s pore diameter ratio to cement particle size is the smallest. This hinders the migration of Grade 42.5 Portland Cement particles through its pore channels, resulting in a maximum filtration coefficient. In contrast, the S3 sand sample exhibits the largest pore diameter ratio to cement particle size. It facilitates more efficient migration of 1250 mesh superfine cement particles through its pore channels, leading to a minimum filtration coefficient. It suggests that as the ratio of pore diameter to cement particle size increases, fewer cement particles are filtered and retained, resulting in a lower filtration coefficient.

### 4.4. Influence of Slurry Density

When the water–cement ratio is 1:1, the density *ρ*_g_ of Grade 42.5 Portland Cement slurry is 1.45 g/cm^3^, and the volume fraction of cement particles *φ* is 0.275; for the 800 mesh superfine cement slurry, the density is 1.46 g/cm^3^ and *φ* = 0.27; and for the 1250 mesh superfine cement, the density reaches 1.48 g/cm^3^ and *φ* = 0.26. This suggests that under an identical water–cement ratio, the smaller the cement particle size, the larger the specific surface area, and the prepared cement slurry shows higher density but lower fluidity. This is why the slurry diffusion distance in the S2 and S3 sand samples is shorter under the same grouting conditions.

## 5. Discussion

1. This study establishes a time-dependent filtration–diffusion coupling model to characterize grout performance degradation in sandy media quantitatively. The feedback effect between particle filtration and slurry diffusion reveals the mechanism of nonlinear attenuation of slurry density along the diffusion distance, which cannot be realized by the static *λ* model [35,36,37,38].
2. We experimentally verified the grout density attenuation law under filtration through grouting experiments. The results demonstrate that the filtration coefficient dynamically varies with cement slurry diffusion distance rather than remaining constant.
3. The emergence of flow instabilities—characterized by the amplification rather than the attenuation of minor disturbances over time—significantly governs filtration efficiency. In our experiments, these instabilities manifest as localized preferential flow channels and intermittent channel clogging. This phenomenon primarily stems from particle retention during filtration, which reduces slurry viscosity through shear-thinning behavior, consequently lowering the Reynolds number and delaying turbulence onset. Furthermore, the heterogeneous pore structure of natural sand amplifies disturbances, while narrow pore throats alter the slurry’s velocity gradient.
4. This study has limitations in both theoretical modeling and experimental design. Firstly, the theoretical framework relies on Darcy’s laminar flow assumption and excludes complex scenarios involving non-Newtonian fluid behavior or unsaturated sand layers. Secondly, experiments were conducted under constant flow rate conditions, which deviate from the dynamic groundwater environments encountered in real-world engineering applications. Future work will investigate slurry diffusion mechanisms under multifactor coupling effects and validate the model’s applicability using field-collected data.

## 6. Conclusions

1. Along the diffusion distance of the cement slurry, the filtration coefficient in the S1 sand sample decreased from 18.17 × 10^−3^s^−1^ to 1.24 × 10^−3^s^−1^; in the S2 sand sample, it declined from 8.86 × 10^−3^s^−1^ to 3.29 × 10^−3^s^−1^; and, in the S3 sand sample, it reduced from 7.15 × 10^−3^s^−1^ to 1.86 × 10^−3^s^−1^. This illustrates that the filtration coefficient varies dynamically along the diffusion distance of the cement slurry rather than remaining constant. Based on the time-dependent filtration–diffusion coupling model and the maximum filtration coefficient, the time when the pores on the surface of the sand layer are completely blocked can be inverted to control the grouting process in the sand layer reinforcement project.
2. When the pore channel of the S1 sand sample is fully obstructed, the filtration coefficient is 6.3 × 10^−3^s^−1^, corresponding to a surface filtration range of 0–30 cm; for the S2 sand sample, with its pore channel fully obstructed, the filtration coefficient is 15.27 × 10^3^s^−1^, and the surface filtration range is 0–10 cm; similarly, for the S3 sand sample, where its pore channel entirely blocked, the filtration coefficient measures 12.29 × 10^−3^s^−1^ alongside a corresponding surface filtration range of 0–10 cm.
3. Under a water–cement ratio of 1:1, the diffusion distance of the Grade 42.5 Portland Cement slurry in the S1 sand sample is measured at 190 cm. In contrast, the diffusion distances for the 800 mesh superfine cement slurry in the S2 sand sample and the 1250 mesh superfine cement slurry in the S3 sand sample are recorded as 69 cm and 87 cm, respectively. The longer the range of surface filtration in the sand sample, the farther the final diffusion distance of the slurry. At the same time, the spacing of boreholes in the sand layer reinforcement project can be optimized based on the surface filtration range and the final diffusion distance.
4. As the ratio of sand pore diameter to cement particle size increases, the adsorption and obstruction effects of the medium skeleton on cement particles diminish, leading to a reduced amount of retained particles and a smaller filtration coefficient. Under a constant water–cement ratio, smaller cement particle sizes result in a higher density of the prepared cement slurry but lower fluidity. These factors significantly affect the diffusion of cement slurry within the sandy medium.

## Figures and Tables

**Table 1:** Particle size distribution of sand.

Sand Sample	Mass of Particles with Different Graded Particle Sizes (g)						Fineness Modulus	Type
	4.75–2.36 mm	2.36–1.18 mm	1.18–0.6 mm	0.6–0.3 mm	0.3–0.15 mm	<0.15 mm		
S1	0	251.8	181.8	61.2	3.0	2.2	3.4	Coarse sand
S2	0	151.7	167.8	171.5	4.8	4.2	2.9	Medium sand
S3	0	102.6	131.8	246.2	9.4	10.0	2.6	Medium sand

**Table 2:** Structural parameters of the sand samples.

Sand Sample	*n*_0_	*D*_0_/mm	*d*_01_/mm	*d*_02_/mm	*d*_03_/mm
S1	0.388	1.27	0.70	0.62	0.54
S2	0.338	0.99	0.48	0.42	0.41
S3	0.329	0.83	0.38	0.34	0.36

**Table 3:** Performance parameters of cement grout.

Material Type	Water–Cement Ratio	*ρ*_g_ (g/cm^3^)	Viscosity *μ*_1_ (mPa·s)	Initial Setting Time (min)	*φ*	Bleeding Rate (%)
P. O. 42.5	1:1	1.45	4.53	896	0.275	26.0
800 mesh superfine cement		1.46	5.87	763	0.270	9.0
1250 mesh superfine cement		1.48	6.96	630	0.260	2.0

Note: The test temperature is maintained at 20 °C, and all measurement data are averaged.

**Table 4:** Grouting test scheme.

Number	Sand Sample	Cement Type	Water–Cement Ratio of Slurry
1	S1	P. O. 42.5	1:1
2	S2	800 mesh superfine cement	
3	S3	1250 mesh superfine cement	

**Table 5:** S1 sand sample—Grade 42.5 Portland Cement experimental results.

Position /cm	*m*_TTL_ /g	*v*_TTL_ /cm^3^	*ρ*_g_ g/cm^3^	w:c	*m*_w_ /g	*m*_c_ /g	*v*_c_ /cm^3^	*φ*	*λ* 10^−3^·s^−1^
10	102.9	76.2	1.35	1.18:1.00	55.7	47.2	20.5	0.269	18.17
30	102.1	76.2	1.34	1.21:1.00	55.9	46.2	20.3	0.266	6.27
50	101.5	76.4	1.33	1.25:1.00	56.4	45.1	20.0	0.262	3.86
70	98.0	76.2	1.29	1.43:1.00	57.6	40.4	18.6	0.244	3.03
90	94.7	76.1	1.24	1.74:1.00	60.2	34.5	15.9	0.209	2.42
110	94.5	76.2	1.24	1.74:1.00	60.0	34.5	16.2	0.213	2.06
130	94.0	76.2	1.23	1.88:1.00	61.4	32.6	14.8	0.194	1.56
150	93.0	76.8	1.21	2.03:1.00	62.3	30.7	14.5	0.189	1.51
170	92.3	76.2	1.21	2.03:1.00	61.8	30.5	14.4	0.189	1.24

Note: (1) *m*_TTL_ is the total mass of the effluent slurry; *v*_TTL_ is the total volume of the effluent slurry. (2) *λ* is calculated using Equation (14).

**Table 6:** S2 sand sample—800 mesh superfine cement experimental results.

Position /cm	*m*_TTL_ /g	*v*_TTL_ /cm^3^	*ρ*_g_ g/cm^3^	w:c	*m*_w_ /g	*m*_c_ /g	*v*_c_ /cm^3^	*φ*	*λ* 10^−3^·s^−1^
10	91.6	66.4	1.38	1.22:1.00	50.3	41.3	16.1	0.242	8.86
30	84.1	66.2	1.27	1.87:1.00	54.8	29.3	11.4	0.172	2.74
50	39.5	33.2	1.19	2.43:1.00	28.0	11.5	5.2	0.157	3.29

Note: (1) *m*_TTL_ is the total mass of the effluent slurry; *v*_TTL_ is the total volume of the effluent slurry. (2) *λ* is calculated using Equation (14).

**Table 7:** S3 sand sample—1250 mesh superfine cement experimental results.

Position /cm	*m*_TTL_ /g	*v*_TTL_ /cm^3^	*ρ*_g_ g/cm^3^	w:c	*m*_w_ /g	*m*_c_ /g	*v*_c_ /cm^3^	*φ*	*λ* 10^−3^·s^−1^
10	87.5	64.6	1.35	1.38:1.00	50.7	36.8	13.9	0.215	7.15
30	84.4	64.4	1.31	1.57:1.00	51.6	32.8	12.8	0.20	2.89
50	79.0	64.6	1.22	2.19:1.00	54.2	24.8	10.4	0.161	2.46
70	76	64.6	1.18	2.76:1.00	55.8	20.2	8.8	0.136	1.86

Note: (1) *m*_TTL_ is the total mass of the effluent slurry; *v*_TTL_ is the total volume of the effluent slurry. (2) *λ* is calculated using Equation (14).

**Table 8:** Filtration coefficient of previous studies [34,35,36,37,38].

Literature	Suspended Particles		Porous Media		Particle Size Ratio	Filtration Coefficient *λ* 10^−3^·s^−1^
	Type	Particle Size /μm	Type	Particle Size /μm		
[34]	Polyvinyl chloride	1.3	Glass microspheres	460	353.8	2.90
				425	326.9	6.60
			Sand	600	461.5	6.16
[35]	Polystyrene	900	Plastic balls	12500	13.9	0.99
		1060			11.8	1.48
[36]	Corner quartz	<20	Glass microspheres	2000	<100	3.70
	Polystyrene	65		2000	30.8	17.20
		90			22.2	35.00
		125			16	47.88
[37]	Colibacillus	1	Sand	104	104	2.74
				227	227	0.52
	Seaweed	15		829	55.3	3.10
[38]	Iron hydroxide	4–25	Sand	700	28–175	0.84

Note: The particle size ratio is the porous media particle size divided by the suspended particle size.

**Table 9:** The ratio of pore diameter to cement particle size.

Sand Sample	*d*_01_/Cement Particle Size	*d*_02_/Cement Particle Size	*d*_03_/Cement Particle Size	Filtration Coefficient *λ* (10^−3^s^−1^)		
				10 cm	30 cm	50 cm
S1	41	36	32	18.17	6.27	3.86
S2	69	60	59	8.86	2.74	3.29
S3	95	85	90	7.15	2.89	2.46

## Data Availability

The raw data supporting the conclusions of this article will be made available by the authors upon request.
